# Chemical Speciation of Aluminum in Wine by LC–ICP–MS

**DOI:** 10.3390/molecules25051069

**Published:** 2020-02-27

**Authors:** Katarzyna Karaś, Anetta Zioła-Frankowska, Marcin Frankowski

**Affiliations:** 1 Department of Analytical and Environmental Chemistry, Adam Mickiewicz University Poznań, Faculty of Chemistry, Uniwersytetu Poznańskiego 8, 61–614 Poznań, Poland; katarzyna.karas@amu.edu.pl; 2 Department of Analytical Chemistry, Adam Mickiewicz University Poznań, Faculty of Chemistry, Uniwersytetu Poznańskiego 8, 61–614 Poznań, Poland; anettazf@amu.edu.pl

**Keywords:** speciation analysis, aluminum, organic and inorganic complexes of aluminum, wine, LC–ICP–MS

## Abstract

Aluminum is very common in the natural environment and in everyday human life. We are living in the “aluminum age.” Its average daily intake should not exceed a few mg/day. Unfortunately, despite the growing number of alarming data about the toxicity of this element, human exposure to aluminum is constantly increasing. The toxicity and bioavailability of aluminum depends mainly on the form in which it occurs. The main variables conditioning the form are the concentration, the type, the molar ratio of aluminum to ligand, the pH value, and the temperature. This research presents a new method for speciation analysis of both inorganic and organic aluminum complexes in model solutions by LC–ICP–MS. Different solutions with variable pH values and different Al/ligand molar ratios (fluorides and several organic ligands, e.g., citrates and oxalates ions) were used. The chromatographic separation process was carried out based on isocratic and gradient elution, using a cation exchange analytical column. All determinations have been confirmed based on chemical equilibrium modeling programs. The new developed method was successfully applied for the first time in speciation analysis of real samples: white and red wine.

## 1. Introduction

Aluminum is present mostly in commonly consumed beverages, such as water, juices, tea, and alcohol (wine and beer). The average total daily dietary intake of aluminum is a few mg/day [1]. Direct contact with Al is also present during food processing, packaging, and storing (such as kitchen foil, vessels, and various food additives), as well as during dermal applications of personal-care products [2,3,4]. Human exposure to Al is also rising because of acid rains, which cause the partial dissolution of soil aluminum, and because of the presence of Al in tap and drinking waters due to the flocculants used in water treatment plants [5].

Toxicity of metal depends on the upon of the absorbed dose, the route of exposure and also duration of exposure [6]. Aluminum has an active role in some neurodegenerative diseases, such as amyotrophic lateral sclerosis or Alzheimer’s and Parkinson’s dementia. [2,7,8,9,10,11,12,13,14]. The toxicity of Al also has an effect on mineral nutrient uptake and the composition in plants. It is interesting that aluminum accumulation in different morphological parts of plants and leaves is an individual mechanism by given plant species [15,16,17].

The mobility, toxicity, and bioavailability of aluminum mainly depend on the form in which this element appears [18]. Other factors such as pH value, the type of a ligand, temperature, and reaction time also have effects on aluminum chemistry [14]. The most toxic form of this elements for living organisms is inorganic aluminum—Al^3+^, AlOH^2+^, Al(OH)_2_^+^, and so on [19,20]. Aluminum can also bond with organic substances—fulvic, humic, malic, tartaric acids, sugars, etc. [21]. It should be mentioned that malic and citrate acids are interesting for aluminum in plants, due to the specific form in which they occur and are toxic in different conditions. It is especially important for the detoxification process of aluminum ions [22].

Speciation of aluminum is key for assessing its toxicity and bioavailability [2]. However, there are still problems with this step associated with the availability of reference substances, solutions, and methods, which can occur in a system of chromatographic distribution and can change or degrade aluminum forms [23]. Other difficulties are the participation of aluminum species during various chemical reactions, low concentrations, and usually complex matrices, which are significant for analytical detection systems [19].

Among the available analytical methods used to identify particular forms of Al, chemical modeling as an indirect method and a combination of liquid chromatography and spectrometry detection as the best direct method can be distinguished. Various computer software can provide necessary information for calculations of Al species in terms of pH, ionic strength of solution, and temperature. Well-known models have been developed for speciation of aluminum, e.g., MINEQL, WHAM, ALCHEM and GEOCHEM, SOLMI, NEQ88, MINTE, KRIMAT, and SIMPLISIMA [4,23,24,25,26].

The total amount of aluminum can be measured by different analytical techniques, such as atomic absorption spectrometry (AAS), atomic emission spectrometry (AES), mass spectrometry (MS), inductively coupled plasma (ICP), and even spectrophotometry after digestion. It is well-known that hyphenated techniques are the most effective methods for the speciation and determination of Al species in biological and environmental systems [24,25]. Using an HPLC–ICP–MS can yield high sensitivity, selectivity in a wide linear dynamic range, and low detection limits, which are necessary to perform correct quantitative and qualitative analysis [18,27,28,29]. Nevertheless, using this type of analytical system is associated with problems related to mobile phases, which contain organic solvents or high salt content. These factors can cause a clogging of the torch or nebulizer and a quenching of plasma, which is problematic [18].

Wine is the most widely consumed alcoholic beverage in many parts of the world. The law directly defines every step in the winemaking process. It is very important to obtain data, including microbiological, chemical, and physiological changes, which can occur during grape manufacturing and wine production. Analytical measurements are necessary to ensure safety and quality control [30,31,32]. From a chemical point of view, wine consists of a water–ethanol mixture with a large number of organic and inorganic compounds. The content and different kinds play a significant role in the taste, aroma, and color [31,33,34,35]. The quantity of these compounds is related to the grape variety, the type of soil, the climatic condition, and the impurities during the growth of fruits [36]. Due to such a complex matrix, the best solution for defining the toxicity of wine samples is to perform speciation analysis, using a proper analytical technique.

The main aims of this research was to (1) develop a new method for the separation and speciation analysis of aluminum and aluminum complexes by LC–ICP–MS, as a direct method for the determination of toxic forms of aluminum (Al^3+^ and Al–inorganic, mainly AlF _(x=2,3,4)_^(3-x)^) and as an indirect method for Al–organic complexes; (2) confirm data from the analytical system with a computer chemical modeling program; and (3) apply the developed method to the speciation analysis of aluminum in selected white and red wine samples from Polish vineyards.

## 2. Results and Discussion

### 2.1. Method Development for Speciation Analysis of Aluminum

Based on the previous studies conducted by Milaćić et al. (1998), to elute an Al^3+^ form from a column, a high concentration of mobile phase (NH_4_NO_3_) is needed [37]. Separation was performed based on gradient elution, with the use of a cation exchange analytical column. During the analysis, different gradients were checked. It was decided that a final experiment using 10 and 500 mM of NH_4_NO_3_ gradient elution at pH = 3.00 ± 0.01 would be carried out.

The first signal characterized by t_R_ = 6.05 min comes from gradient elution separation mode. The second one, characterized by t_R_ = 7.5 min, represents the Al^3+^ form. During the separation process, a precipitation of eluent salt was observed. The consequence of this process was a clogging of the torch. This was also observed by Bayón et al. (1998) and by Zioła-Frankowska et al. (2015) [18,38]. This problem was solved by using a bubbler (argon humidifier) in line of the carrier gas in ICP–MS (Shimadzu, Japan). The application of bubbler causes humidification of the carrier gas (argon), which prevents salt formation and therefore torch clogging. No effects on determination, using the bubbler, were observed. However, other problems—a switching off of the plasma during analysis, the necessity of cleaning the cones after a few hours (analysis time 8 min), and an additional signal from the gradient elution—were observed. To avoid these effects, isocratic elution was chosen for further analysis.

Before that, one more aspect was checked. It was assumed that, if a standard solution is dosed to the column, the isocratic elution should not give a signal from the Al^3+^ form in the system with the column. In the next stage, the column was removed. The conducted process confirmed the total elution of the Al^3+^ form from the chromatography column.

**Figure 1:**
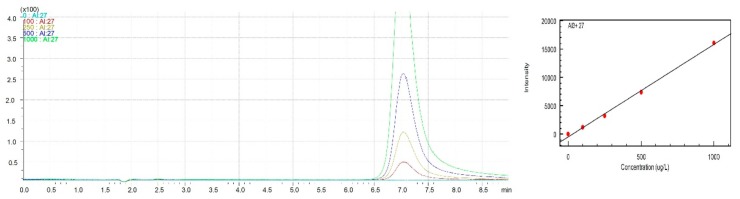
The overlapped chromatograms of Al standards solutions for HPLC–ICP–MS with the use of Hamilton PRP-X200 analytical column and calibration curve of aluminum standard solutions–isocratic elution.

Afterward, the isocratic elution process was conducted. The concentration of the NH_4_NO_3_ mobile phase was set to 25 mM. Figure 1 presents the obtained overlaid chromatograms of the aluminum standards solutions 100/250/500/1000 (µg L^−1^) for HPLC–ICP–MS, the calibration curve of aluminum at pH = 3.0 for isocratic elution, and the calibration curve of aluminum at pH = 3.0 for aluminum standards solutions 50/100/250/500/1000 (µg L^−1^) for HPLC–ICP–MS.

### 2.2. Models vs. Standard Solution Analysis

Model solutions were prepared, to check the occurrence and stability of different organic and inorganic complexes of aluminum, depending on the Al/ligand stoichiometry and pH value. The separation of aluminum complexes and the Al^3+^ form was conducted in a single analytical cycle. In every prepared model solution measured on the HPLC–ICP–MS system, the concentration of Al was equal to 0.25 (mg L^−1^), as in previous studies conducted by Frankowski et al. (2015), with a variable concentration of ligands, which corresponded to the appropriate Al/ligand molar ratio: 1:0.5, 1:1, 1:10, and 1:100 [18].

#### 2.2.1. Aluminum Fluoride Complexes

**Figure 2:**
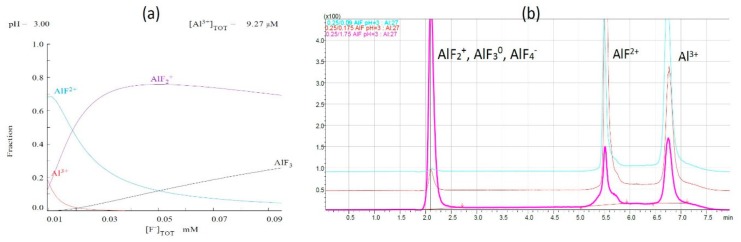
(**a**) Data from chemical modeling program for 1:1 and 1:10 Al/F molar ratio and (**b**) overlapped chromatograms Al/F for 1:0.5, 1:1, and 1:10 molar ratio.

All solutions prepared for HPLC–ICP–MS analysis had a constant Al concentration (0.25 mg L^−1^) and pH value (3.0), but had variable concentrations of fluoride ligand. The results obtained from the chemical modeling are presented in Figure 2a. The theoretical calculations suggest that, with the increases in fluoride concentrations, the amount of AlF_2_^+^ and AlF_3_ forms also increases, but the amount of AlF^2+^ and Al^3+^ forms decreases. The data are presented in Figure 2b.

Based on the analysis of the aluminum fluoride complexes solutions, the molar ratios of Al/F were 1:0.5, 1:1, and 1:10. The pH was equal to 3.0. The observed individual form was eluted in the following order:

First signal: AlF_2_^+^, AlF_3_^0^, and AlF_4_^-^, t_R_ = 2.1 min,

Second signal: AlF^2+^; t_R_ = 5.5 min,

Third signal: Al^3+^; t_R_ = 6.8 min.

The overlapped chromatograms of specified stoichiometry of Al/F are presented in Figure 2b. The obtained results comply with the theoretical calculations from the Mineql chemical program. Together with the increase of the fluoride concentration, the presence of the first form increases, but the presence of the second form and Al^3+^ form decreases, which means that most of the aluminum formed complexes. Based on the chromatograms, forms of the aluminum/fluoride complexes were evaluated according to the charge. The dominant aluminum fluoride form was the first one (AlF_4_^−^, AlF_3_^0^, and AlF_2_^+^). Increasing the fluoride concentration in relation to the molar ratio caused the total domination of fluoride aluminum complexes with a charge of +1, 0, and −1, which means there was a total complexation of free forms of aluminum Al^3+^ with fluoride. Moreover, in the case of an Al/F 1:10 molar ratio, no Al^3+^ was observed. This is also confirmed by previous results obtained by Frankowski et al. [18,26,39,40].

#### 2.2.2. Aluminum Organic Complexes

It should be pointed out that the research was conducted to create a new method that allows one to determine not only toxic forms of aluminum (Al^3+^ and Al–inorganic complexes) but also organic forms, which are the dominant form and complex of aluminum, and these have not yet been directly determined by using HPLC–ICP–MS, especially in these types of samples. The analysis allows for the separation of Al/citrate and Al/oxalic complexes. In the case of other complexes, they were most likely depredated.

#### 2.2.3. Al/Citrate

**Figure 3:**
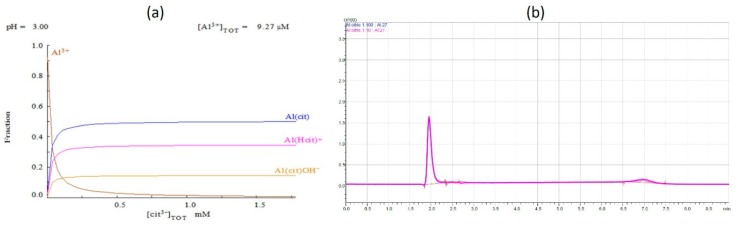
(**a**) Data from chemical modeling program for Al/citrate 1:1 and 1:100 molar ratio and (**b**) overlapped chromatograms for Al/citrate 1:10 and 1:100 molar ratio.

Aluminum citrate species are known as important complex components in biological systems, for example, for detoxification aluminum ions, because they use citric acid as a complexing reagent [25,27]. According to data from the chemical modeling equilibrium program presented in Figure 3a, at a constant pH, the Al/citrate forms Al(cit)OH^−^ (charge = −1), Al(cit) (charge = 0), and Al(cit)H^+^ (charge = +1) increase together with increasing concentrations of citrate ligands, while the presence of free Al^3+^ decreases. The overlapped chromatograms shown in Figure 3b pointed to two signals. The first one is very weak and responds to the −1, 0, and +1 form of aluminum citrate complexes, and the second one is Al^3+^. Interestingly, the free Al^3+^ signal suffers from broadening. This was also observed by Happel and Seubert (2006) during the characterization of stable aluminum-citrate species by ion chromatography. During analysis of compound [Al_3_cit_2_H_−2_]^−^, it was found that the large size of the compound causes the delocalization of a negative charge. In the results, the interaction between the compound and functional groups in the column was reduced, which caused the small resolution of the obtained peak [23]. This was also confirmed by Chen et al. (2010), who checked the effect of mobile phase flow for separation Al-complex compounds [8]. They assumed that the resolution of the two chromatographic peaks of Al-citrate and Al-transferrin obtained in HPLC analysis became weak when the flow rate of mobile phase was higher than 0.7 mL min^−1^. Further confirmation may come from Cardiano et al. (2017), who proved that, together with the increasing molecular weight, the number of charges grows and, as a result, the interaction with metal cations also increases [21]. Additionally, the presence of the –OH group increased the possibility of interaction between Al^3+^.

#### 2.2.4. Al/Oxalate

**Figure 4:**
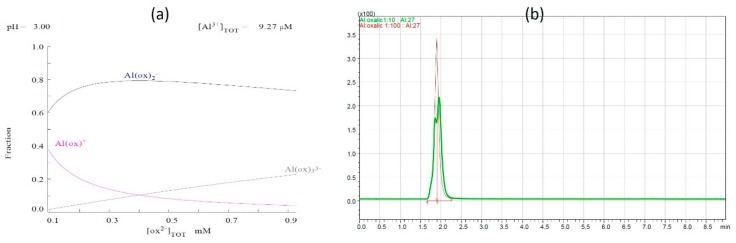
(**a**) Data from chemical modeling program for Al/oxalate 1:10 and 1:100 molar ratio and (**b**) overlapped chromatograms for Al/oxalate 1:10 and 1:100 molar ratio.

In this case, the proportion of the complexed form increased with the increase in ligand concentration, and the share of the free and toxic aluminum form Al^3+^ decreased. The higher the ligand concentration was, the more distinctly weak the separated peak of the first separated form of the Al/oxalate form was (the sum of Alox^−1^ and Alox^+1^ form). The share of individual forms changes when the concentration of the ligand is rising. In the case of a molar ratio of 1:100, the dominant forms are the −1 and –3 forms. We noticed the absence of the Al^3+^ form, which suggests the total complexation of aluminum, and this is presented in Figure 4a,b.

The study conducted by Borrmann and Seubert (1996) conducted with 1:1 Al–Ox suggested a non-charged species or a negatively charged species, i.e., AlOx_2_ or AlHOx_2_^−^. The first one is more probable because of the higher value of its stability constant. The chemical modeling conducted in this study confirmed the presence of the negatively charged form of Al/oxalate complex—Alox^−1^. A possible explanation mentioned in the previous study for the division of non-charged species and negatively charged species of Al–Ox systems by a cation exchange column is the presence of an additional separation mechanism, along with the sheer ion replacement. It is most likely connected to the competing of ligands [41].

### 2.3. Wine Samples

#### 2.3.1. Total Content of Aluminum, Fluorides, and Organic Ions

The results obtained from ICP–MS analysis showed a higher content of aluminum in white wines in comparison to red wines. The highest value was noted for white wines, and it was for Wine No. 9—2.64 mg L^−1^ (Hibernal from the Chodorowa winery) and Wine No. 10—2.38 mg L^−1^ (Seyval Blanc also from the Chodorowa winery). The highest values in red wine were noted for Wine No. 26—0.45 mg L^−1^ (Pinot Noir from the Srebrna Góra winery) and Wine No. 29—0.52 mg L^−1^ (Pinot Noir from the Adoria winery). In addition, the smallest values in white wines were noted for Wine No. 4—0.05 mg L^−1^ (Cytryn from the Patria winery) and for Wine No. 8—0.13 mg L^−1^ (Sibera from the Kępa Wislicka winery). In comparison to red wines, the smallest value was almost on the same level and was noted for Wine No. 5—0.11 mg L^−1^ (Koral from the Patria winery) and Wine No. 21—0.13 mg L^−1^ (Regulus B from the De Sas winery). All results are presented in Table S2.

The obtained results are comparable with other data from different research work based on Al levels in alcohol beverages using other analytical methods of analysis. Lorenzo et al. (1998) determined Al in 70 different samples of alcoholic beverages widely consumed in Spain. The value range for white wines was 0.189–1.683 mg L^−1^ and for red wines was 0.072–1.254 mg L^−1^. Additionally, the level of Al in the selected wines from other different countries was checked: France: 0.25–2.55 mg L^−1^; Germany: 0.63–1.120 mg L^−1^; and Italy: 0.089–1.463 mg L^−1^. The study was performed by using the GFAAS method [3]. Another researcher, Tariba (2011), defined the concentration of Al in selected wine samples from different countries by ICP–OES: Argentina: 0.017–0.018 mg L^−1^; Czech Republic: 0.132–1.665 mg L^−1^; Croatia: 0.244–0.809 mg L^−1^; Greece: 0.36–9.5 mg L^−1^; and Hungary: 0.01–1.5 mg L^−1^ [32]. Cabrita et al. (2018) checked the concentration of several elements in Portuguese wines. The average concentration of Al in white wines determined by ICP–MS was 0.447 mg L^−1^, where the value range was 0.125–0.988 mg L^−1^; in red wines, it was 0.421 mg L^−1^, where the value range was 0.140–1.488 mg L^−1^. In this study, ICP–MS was used [42]. Frankowski and Zioła-Frankowska (2016), in determination of metals and metalloids in wine, using ICP–OES and a mini-torch, determined the highest concentration of aluminum in white wines—$1.07_{-}^{\;+\;}$0.01 mg L^−1^—and a lower concentration in red wines, which amounted to 0.6 mg L^−1^ [43].

The obtained data from the determination of the total content of aluminum by ICP–MS, as well as the sum of aluminum forms by LC–ICP–MS, are comparable and are presented in Supplementary Table S2.

Results obtained from the Fluoride Ion Selective Electrode (FISE) method analysis showed a higher content of fluorides in white wines in comparison to red wines. The highest value was noted in Wine No. 31—0.257 mg L^−^^1^ (Solaris from the Turnau winery) and Wine No. 3—0.21 mg L^−1^ (Cymbały from the Sztukówka winery). The highest value for red wine was noted in Wine No. 23—0.197 mg L^−1^ (Cabernet Cortis from the Srebrna Góra winery). The smallest value in white wine was noted in Wine No. 7—0.027 mg L^−1^ (Passage Cuvee from the Equus winery); in red wines, it was noted in Wine No. 6 on the same level—0.027 mg L^−1^ (Magnesia Prestige from the Equus winery). The average concentration of fluorides in this study was 0.12 mg L^−1^. The obtained results are comparable with data obtained by other researchers. In a study conducted by S. Paz et al. (2016), the average concentration of fluorides was 0.13 mg L^−1^. During the analysis of 23 wine samples, the smallest value was 0.03 mg L^−1^, but the highest was 0.35 mg L^−1^ [44]. Rodriguez Gómez et al. (2003) obtained an average value equal to 0.15 mg L^−1^. In their research, they cited results obtained by other authors, such as Rincon and Cabeza (0.25 mg L^−1^), Hardisson (0.2 mg L^−1^), and De Baenst et al. (0.17 mg L^−1^) [45].

For comparison of the total content of organic ions (acetate, citrate, formate, lactate, and oxalate) with the total content of aluminum, three white wines (No. 15, Daromini Blanc from the Płachockich vinery, No. 28, Chardonnay from the Adoria vinery, and No. 31, Solaris from the Tournau vinery) and three red wines (No. 1, Pinore XII from the Jaworek vinery, No. 21, Regulus B from the De Sas vinery, and No. 24—Rondo from the Srebrna Góra vinery) were chosen. In each analyzed wine, the concentration of organic ions was significantly higher than the concentration of aluminum. The average obtained results for white wines showed that the total content of organic ions was 3450 mg L^−1^, and the average total content of aluminum in the same wines was 0.73 mg L^−1^. The average total content of organic ions in red wines was 3276.5 mg L^−1^, and the average total content of aluminum was 0.2 mg L^−1^. Making a deeper analysis, it can be estimated that the average content of acetates in white wines was 538.9 mg L^−1^, of citrates was 41.7 mg L^−1^, of formates was 743 mg L^−1^, of lactates was 113.4 mg L^−1^, and of oxalates was 875.6 mg L^−1^. The average content of the same ions in red wines was 574.4 mg L^−1^ for acetates, 43.5 mg L^−1^ for citrates, 853.5 mg L^−1^ for formates, 1481 mg L^−1^ for lactates, and 515.5 mg L^−1^ for oxalates. Yun Fa et al. (2018) studied the content of organic acids in wine and also found higher amounts of organic ions in red wine samples in comparison to amounts obtained from the white wine sample. Studies were performed via valve-switching ion chromatography [34].

#### 2.3.2. Speciation Analysis of Wine

**Figure 5:**
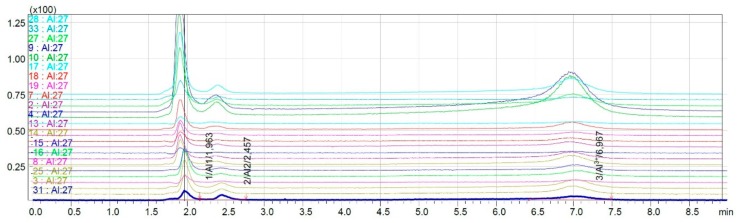
Overlapped chromatograms obtained for analyzed white wine samples.

Speciation analysis of 33 wine samples was carried out, using the developed chromatographic method. The results of 19 white wine samples’ analysis are presented in Figure 5.

The results show the separation of three analytical signals. The retention time of the first one is t_r_ = 2.0 min and is similar to the elution time of the first signals of the aluminum complexes with citrates (t_R_ = 2.0 min) and the aluminum complexes with oxalates (t_r_ = 1.9 min), which suggested the creation of organic types of complexes. The second signal was obtained at a very low level. Based on the retention time, t_R_ = 2.4 min, the second form of AlF^2+^ with a retention time of t_R_ = 2.5 min was expected. The retention time of the third signal was t_R_ = 7.0 min, which corresponded to the retention time of the third form of aluminum—Al^3+^.

**Figure 6:**
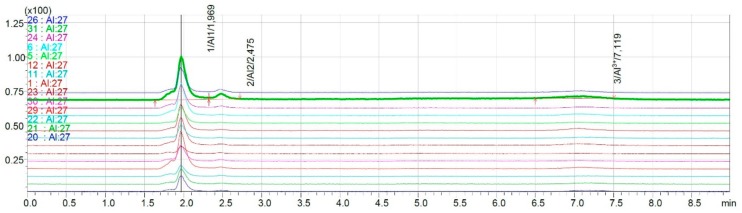
Overlapped chromatograms obtained for analyzed red wine samples.

The results of the analysis of 14 red wines are presented in Figure 6. Three analytical signals were obtained during chromatographic analysis. The retention time of the first one was t_R_ = 1.9 min, which corresponded to the retention time of the first signal of aluminum complexes with fluorides, citrates, and oxalates (I form—the sum of +1, 0, −1). Second signal characterized by t_R_ = 2.5 is defined as the II form—AlF^2+^. The third signal with t_R_ = 7.1 min responds to III form of aluminum—Al^3+^.

**Figure 7:**
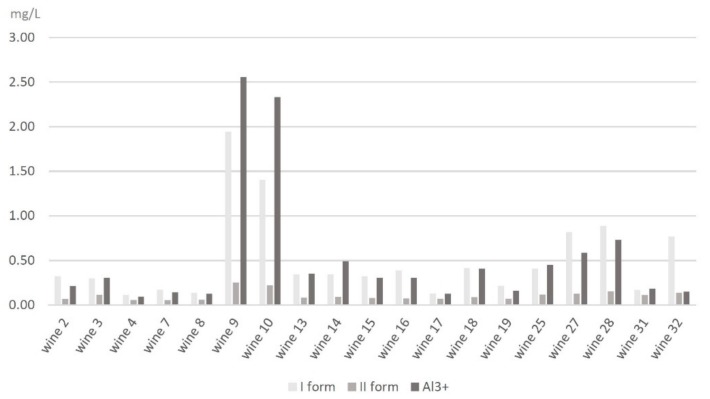
Comparison of contribution all forms of aluminum in white wines.

Based on the value of the peak area, the comparison of the amounts of specific forms of aluminum in white and red wines was performed. The results for white wines show that, for the first form (+1, 0, −1), which includes complexes of aluminum with fluorides, citrates, and oxalates, the largest contribution was 1.94 mg L^−1^ from Wine No. 9 (Hibernal from the Chodorowa winery), followed by 1.40 mg L^−1^ from Wine 10 (Seyval Blanc also from the Chodorowa winery). Similar data were observed for the second signal (AlF^2+^), 0.25 mg L^−1^ in Wine No. 9 and 0.22 mg L^−1^ in Wine No. 10, and the third signal corresponding to the Al^3+^ form, 2.55 mg L^−1^ in Wine No. 9 and 2.33 mg L^−1^ in Wine No. 10. On the other hand, the smallest values for the first form were noted in Wine No. 4 at 0.11 mg L^−1^ (Cytryn from the Patria winery), Wine No. 17 at 0.13 mg L^−1^ (Parus A from the De Sas winery), and Wine No. 8 at 0.14 mg L^−1^ Sibera from the Kępa Wiślicka winery). Data observed for the second signal were 0.05 mg L^−1^ in Wine No. 7 (Passage Cuvee from the Equus winery) and 0.06 mg L^−1^ in Wine No. 4 (Cytryn from the Patria winery) and Wine No. 8 (Sibera from the Kępa Wiślicka winery). The smallest data for the third signal were noted for Wine No. 4—0.09 mg L^−1^. All results are presented in Figure 7.

**Figure 8:**
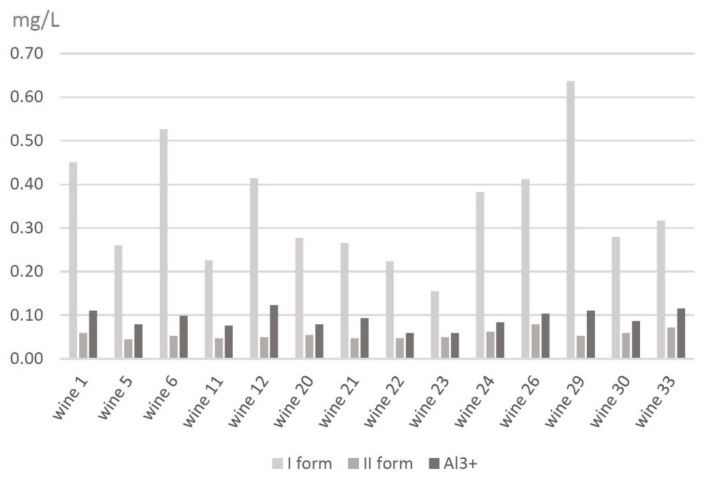
Comparison of the contribution of all forms of aluminum in red wines.

The results for the red wines show that there is no domination like that observed in white wines. However, the largest contribution of the first form of aluminum was noticeable in Wine No. 29—0.64 mg L^−1^ (Pinot Noir from the Adoria winery). Considering the contribution of the second form of aluminum, the largest one was reported for Wine No. 26—0.08 mg L^−1^ (Cuvee from the Srebrna Góra winery). The largest value for the third signal of aluminum was observed in Wine No. 12 at 0.12 mg L^−1^ (Geltrus XIV from the Płochockich winery) and in Wine No. 33 at 0.12 mg L^−1^ (Mozów 2 from the Mozów winery). The smallest value for the first signal was noted in Wine No. 23 at 0.15 mg L^−1^ (Cabernet Cortis from the Srebrna Góra winery), for the second form in Wine No. 5 at 0.04 mg L^−1^ (Koral from the Patria winery), and for the third form in Wine No. 22 and 23 at 0.06 mg L^−1^ (both from the Srebrna Góra winery). All results are presented in Figure 8.

Based on the chromatographic analysis of the white and red wines, the total content of aluminum complexes is higher in white wines than in red wines. In comparison, the average content of the I form of aluminum in white wines is 0.51 mg L^−1^; in red wines, it is 0.34 mg L^−1^. The average content of the II form in white wines is 0.11 mg L^−1^; in red wines, it is 0.06 mg L^−1^. The average content of the III form in white wines is 0.53 mg L^−1^; in red wines, it is almost six times less—0.09 mg L^−1^. Differences in the distribution of aluminum forms may result from the variety of wine strains, the proportion in which they have been mixed, and the wine regions. Another factor might be the use of different materials during the preparing and storing of wines, including stainless steel, brass containers, plastic tubes and fittings, and oak barrels. These materials can be a source of many elements: Al, Cr, Cd, Co, Fe, Mo, Mn, Ni, Pb, Sr, Ti, V, and Zn [32,46,47,48,49]. An additional factor that might influence the increased level of Al, Cd, Hf, Pb, U, and Zn is bentonite, which is usually added to grape must or finished wine during the clarification process [46,47,49]. For comparison, in the obtained results of previous data from the speciation analysis of aluminum in black and fruity tea conducted by Frankowski (2013) by an HPLC-Fluorescence method, the concentration of each form of aluminum (I, II, and III) in tea is higher than that obtained in wine analysis. However, the concentration of the I form of aluminum (0.505 mg L^−1^) and the II form of aluminum (0.106 mg L^−1^) obtained from white wine analysis is comparable with results obtained for the same forms of aluminum from black tea analysis and is 0.596 mg L^−1^ for the first form and 0.109 mg L^−1^ for the second form of aluminum. The author assumed that the occurrence of forms +1, 0, −1, and +2 was closely related to the aluminum complexes with fluorides, which indicates a higher stability of these complexes in comparison to other inorganic and organic complexes [1]. However, this data obtained from wine analysis and checked by model solution analysis proves that the first signal represents not only aluminum complexes but also complexes with fluorides, citrates, and oxalates, according to +1, 0, and −1 charges. In comparison to another measuring methods, the new method developed for aluminum speciation is less time-consuming and allows for the determination of many more forms of aluminum. Magnier et al. (2014), who used competitive ligand exchange-adsorptive stripping voltamperometry for determining the speciation of aluminum in commonly consumed beverages, both filtered and diluted beverages, before analysis. This method, which relies on titration curves, was time-consuming and required many steps before direct analysis could take place [2]. Based on speciation analysis of iron, being similar to aluminum, different studies were conducted. Lopez-Lopez et al. (2015) successfully conducted molecular absorption spectrophotometry to simplify the direct determination of Fe(II) and the total amount of Fe in wine samples. The proposed method, using 2,2′-dipyridyl ketone picolinoylhydrazone (DPKPH) as a colorimetric reagent, was found to be a fast and simple method that reduces operation times and costs [50]. Ferreira et al. (2019) also proposed a method for speciation analysis of iron in wines. The new approach used combinations of colorimetric reactions between iron(II) and 1,2-otho-phenantholine and used computer vision. However, the proposed method allows for the quantification and speciation of inorganic iron [51]. Ebeler et al. (2019) focused on arsenic in wines, another metal similar to aluminum, and confirmed that the total arsenic and arsenic-species level is variable and dependent on a number of factors, such as origin, winemaking practices, and wine styles [52].

In comparison to the acceptable concentration limit of aluminum in drinking water established by WHO, which is 0.2 mg L^−1^, the obtained content results for wines significantly exceed this limit, which should concern wine consumers [53]. The average volume of a glass of wine is 175 mL. According to this, the average dose of aluminum introduced with a glass of wine to an organism, calculated using the obtained results, is 0.17 mg for white wine and 0.06 mg for red wine. According to the global status report on alcohol and health from 2014, worldwide consumption of alcohol in 2010 was 6.2 L per person aged 15 years or older. The data show that 8.0% of total recorded alcohol is consumed in the form of wine. It represents a fourth of the total consumption of wine in the WHO European Region (25.7%) and a ninth of the total consumption in the WHO Region of the Americas (11.7%), notably due to the high share of wine consumption in Argentina and Chile [54]. By simple calculation, it can be assumed that one statistical person can consume approximately 0.23 mg of aluminum per year by drinking only wine. According to a special report prepared by the Central Statistical Office in Poland, the average consumption of wine in 2016 was equal to 6 L per year [55]. By calculations using the obtained results, it can be assumed that one statistical Polish man can consume approximately 5.8 mg of aluminum by drinking white wine and approximately more than 1.9 mg by drinking red wine during the year. However, this does not exceed the allowable dose of aluminum and is not hazardous to health. Noteworthy is the fact that the Chodorowa winery, which is often noted as having the highest values of aluminum, is located in the south of Poland, in Krakow. According to the report prepared by the Provincial Inspectorate for Environmental Protection in Krakow, based on data collected in 2010–2015, most of the arable soils of this area are considered very acidic (41.2%), acidic (23.5%), or slightly acidic (11.8%). The rest of the soils are neutral. In comparison to earlier years, the share of slightly acidic soils dropped significantly, and the share of very acidic soils significantly increased. At pH values below 4.5 (very acidic), soluble forms of aluminum appear in the soil solution, which damages roots and impairs the uptake of water and nutrients by plants, thus limiting the quantity and quality of crops. In addition, at such a low pH, many harmful elements contained in the soil are mobilized and consumed by plants (including toxic trace elements) [56]. This may explain the high aluminum content in wine samples from the Chodorowa winery.

## 3. Materials and Methods

### 3.1. Analytical System

An ICPMS-2030 Inductively Coupled Plasma Mass Spectrometer (Shimadzu, Japan) directly coupled with a Prominence LC 20Ai inert system was used for the speciation of aluminum. TRM (Time Resolved Measurement) software for LC–ICP–MS was used for controlling both ICP and LC analytical systems. The presence of the inert system eliminates the possibility of metal background leaching from components of the mobile phase. What is important is that the inert liquid chromatography system is highly suitable for the speciation analysis of metals, in which the lowest detection limit is obligatory. The inert liquid chromatography is equipped with a binary pump LC 20Ai, a vacuum degasser (DGU 20A3R), an autosampler (SIL 20AC), a heated column compartment (CTO 20AC), and a controller (CBM 20A) (Shimadzu, Kyoto, Japan). A cation-exchange column Hamilton PRPX-200 (analytical column, 150 mm, 2.1 mm i.d., and a particle size of 10 µm, containing a PSDVB/Sulfonate Exchanger) was used. The analysis time was set to 10 min. Ammonium nitrate was used as a mobile phase with a 2 mL/min flow rate, and pH = 3.00 ± 0.01 (by HNO_3_). The chosen eluent prevents aluminum complexes from decomposing and does not compete with ligands. In all measurements, a 200 µL sample loop was used. The ICP–MS operates at 1000 W, with an 8.0 mL min^−1^ argon plasma gas flow, a 0.7 mL min^−1^ Ar carrier gas flow, and a 1.0 mL min^−1^ Ar auxiliary gas flow. The sampling depth was 4.0 mm. Optimized conditions of the collision cell were −30 V of cell gas voltage, 5.0 V of energy filter voltage, and a 6 mL/min cell gas (He) flow rate.

### 3.2. Reagents and Standards

Ultrapure water (< 0.005 µS) obtained from a Milli-Q Direct 8 purification unit system (Millipore, Burlington, MA, USA, Merck) was used to prepare all solutions. Ammonium nitrate with a concentration of 10–500 mM was prepared from salt ammonium nitrate NH_4_NO_3_ (Sigma-Aldrich, USA). Diluted suprapure nitric acid (Sigma-Aldrich, USA) was used to adjust the pH of the mobile phase and the model solutions. All standard solutions used for calibration were prepared by the volume dilution of the aluminum standard solution 1000 mg/L (Merck, Germany), prepared from Al(NO_3_)_3_. Model solutions of fluorides were prepared from a fluoride standard solution of 1000 mg/L (Merck, USA) by volume dilution. Model solutions of selected acids—citric, fumaric, glutaric, glycolic, glyoxylic, humic, malic, malonic, oxalic, phtalic, quinic, succinic, sulfamic, and tartaric acids—were prepared from their salts (Sigma-Aldrich, USA). Model solutions of acetic, butyric, formic, lactic, propionic pyruvic, tannic, and valeric acids were prepared from their liquid solutions (Sigma-Aldrich, USA). To eliminate potential contamination, all glassware and polypropylene storage bottles were kept in HNO_3_ (10% *v/v*), rinsed three times by ultrapure water, and allowed to dry before use.

### 3.3. Total Content of Aluminum, Fluorides, and Organic Ions (Acetate, Citrate, Formate, and Oxalate)

The determination of fluorides was conducted by a method presented by Frankowski et al. (2015), using the FISE method with a TISAB buffer solution to adjust pH and total ionic strength (Thermo, USA) [18].

Determination of organic ions (acetate, citrate, formate, and oxalate) was conducted by an Ion Chromatography method presented by Frankowski (2016), using LC with conductivity detector equipment with a Dionex A22 analytical column with a AG22 guard column (Thermo, USA) by gradient elution analysis [57].

The total content of aluminum in wine samples was determined by using an ICP–MS analytical technique.

### 3.4. Method Development for Speciation Analysis of Aluminum

#### 3.4.1. Modeling and Model Solutions

**Figure 9:**
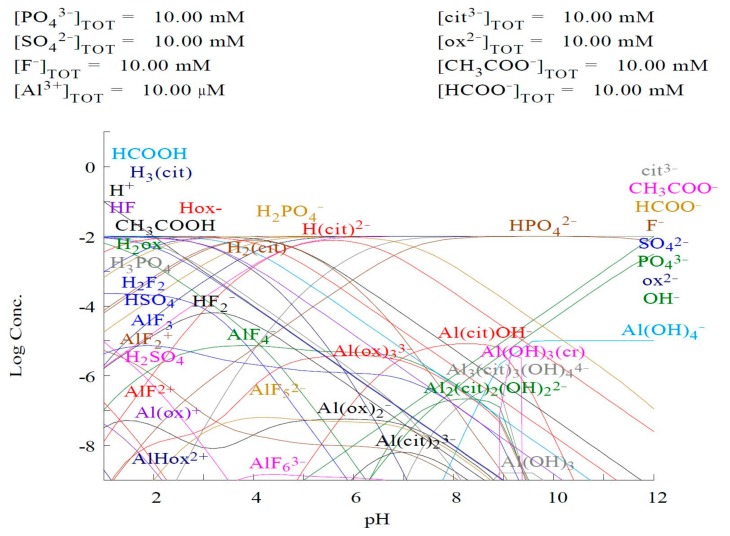
Simple modeling system for constant Al and ligand concentration in function of pH.

The occurrence of certain forms of aluminum species depends on many factors, such as the concentration of aluminum, the type and proportion of ligands, the pH, and the temperature. Figure 9 presents a simple modeling system for a constant Al and a ligand concentration in the function of pH. The chart illustrates the possibility of occurrence of four inorganic forms of aluminum—the hydroxy form, complexes with fluorides, phosphates, and sulfates—and three organic forms of aluminum—complexes with formates, citrates, and oxalates. As is shown, small changes in pH value cause significant differences in the occurrence and contribution of specific forms in solutions. The most important consideration in the anticipation of the mentioned contributions is the competitiveness between ligands, resulting from the value of the stability constants (*logK*) of the Al(III) complexes. In particular, this applies to ions with a high value of *logK*. Each value is obtained from a program designed for chemical modeling called Mineql/Medusa (e.g., complex (logK): AlF^2+^ (7.00); AlF_2_^+^ (12.70); AlF_3_ (16.8); AlF_4_^−^ (19.40); AlF_5_^2−^ (19.40); AlF_6_^3−^ (19.80); Al(cit) (10.05); Al(cit)_2_^3−^ (12.91); Al(cit)OH^−^ (6.53); Al(Hcit)^+^ (12.90); Al_3_(cit)_3_(OH)_7_^7−^ (11.23); Al_2_(cit)_2_(OH)_2_^2−^ (16.56); Al_3_(cit)_3_(OH)_4_^4−^ (19.80); Al(ox)^+^ (7.89); Al(ox)_2_^−^ (13.49); Al(ox)_3_^3−^ (17.32); AlHox^2+^ (7.57) [58].

#### 3.4.2. pH Effect and Adjustment

The pH factor has a major influence on the form of aluminum. The charge of some complexes changes because of the pH dependence of the protonation equilibrium [3]. In addition, the solubility of Al is higher at the lowest value of pH (1–4) and decreases at pH = 8. After this value of pH, the solubility of Al increases again (simulated in the Mineql program). Analyzed model solutions were prepared at 3.0, 3.5, and 4.0 pH values because of the acidic pH of both types of wines. Determinations of pH were conducted, using the Orion 5-star Plus meter (Thermo, USA), with a Single Pore pH electrode (Hamilton, USA).

### 3.5. Wine Samples

It is well-known that the soil composition and geographic origin of grapes have a direct relationship with wine quality [59,60]. It is important to take into account the origin and environmental conditions that contribute to grapes when choosing the appropriate wine strains for analysis. Nineteen white wines and 14 red wines were analyzed. In this group, 28 dry and 5 semi-dry wines were found. Among them, 20 were produced in 2014, 7 in 2015, and 6 in 2013. The samples were analyzed directly and by a volume dilution of 1:10, for both ICP–MS and LC–ICP–MS. The characteristics of wines are presented in Supplementary Table S1.

## 4. Conclusions

Our new method allowed for three separate analytical signals of aluminum complexes (first and second signals) and the Al^3+^ form (third signal). Separation for both inorganic and organic complexes was performed, using a PSDVB-sulfonate exchanger analytical column. Separation of aluminum fluoride, aluminum oxalate, and aluminum citrate complexes with the use of gradient elution allowed for the separation of −3, −1, 0, +1 (1st signal), and +2 (2nd signal) forms, as well as the most toxic aluminum form, Al^3+^, based on model solutions. The obtained results agree with theoretical calculations obtained by using a program for chemical modeling.

In summary, our new method of separation and determination of different forms of aluminum is a good tool for the speciation analysis of wine samples. The suggested method does not cause torch clogging in the ICP–MS spectrometer and determines organic and inorganic forms of aluminum. The new method can be successfully applied in the speciation analysis of samples of a different nature and matrix.

## Supplementary Materials

The following are available online. Table S1: Characteristics of wines, Table S2: Total content of aluminium and the sum of the three signals of aluminium from speciation analysis.
