# Supplementary material for: Chemical Speciation of Aluminum in Wine by LC–ICP–MS
Source: Molecules. 2020 Feb 27;25(5):1069. doi: 10.3390/molecules25051069 (PMC7179188; doi:10.3390/molecules25051069)
Supplement: Supplementary file 1 [file molecules-25-01069-s001.pdf]

Table 1 Supplementary material 1 Characteristics of wines

Vinery	Wine	Sample name	colour	type	wine vintage
ADORIA	Chordonnay	28	white	dry	2014
	Pinot Noir	29	red	dry	2013
	Riesling	27	white	dry	2014
CHODOROWA	Hibernal	9	white	dry	2014
	Rondo	11	red	dry	2015
	Seyval Blanc	10	white	semi-dry	2014
DE SAS	Milvus	19	white	dry	2014
	Parus A	17	white	dry	2014
	Parus B	18	white	semi-dry	2014
	Regulus A	20	red	dry	2015
	Regulus B	21	red	dry	2014
EQUUS	Magnesia Prestige	6	red	dry	2013
	Passage Cuvee	7	white	dry	2013
JAWOREK	Cha-re XII	2	white	dry	2014
	Pinore XII	1	red	dry	2014
PATRIA	Cytryn	4	white	dry	2015
	Koral	5	red	dry	2014
PŁOCHOCKICH	Daromini Blanc	15	white	dry	2015
	Geltus	12	red	dry	2015
	Hibia XIV	16	white	dry	2014
	Lumini XIII	14	white	dry	2014
	Sey Sey	13	white	semi-dry	2015
KĘPA WISLICKA	Sibera	8	white	dry	2014
SREBRNA GÓRA	Cabernet Cortis	23	red	dry	2013
	Cuvee	25	white	dry	2014
	Pinot Noir	26	red	dry	2014
	Regent	22	red	dry	2013
	Rondo	24	red	semi-dry	2013
SZTUKÓWKA	Cymbały	3	white	dry	2015
TURNAU	Cabernet	30	red	dry	2014
	Solaris	31	white	dry	2014
MOZÓW	Mozów 1	32	white	dry	2014
	Mozów 2	33	red	dry	2014

Table 2 Supplementary material 2 Total content of aluminium and the sum of the three signals of aluminium from speciation analysis

Colour	Vinery	Sample Name	sum of the 3 signals of Al. from speciation analysis [mg/L]	Al total[mg/L]
white	JAWOREK Cha-re XIII	wine 2	0,43	0.4
	SZTUKÓWKA Cymbały	wine 3	0,44	0.37
	PATRIA Cytryn	wine 4	0,05	0.05
	EQUUS Passage Cuvee	wine 7	0,25	0.26
	KĘPA WISLICKA Sibera	wine 8	0,15	0.13
	CHODOROWA Hibernal	wine 9	2,58	2.64
	CHODOROWA Seyval Blanc	wine 10	2,47	2.38
	PŁACHOCKICH Sey sey	wine 13	0,41	0.4
	PŁACHOCKICH Lumini XIII	wine 14	0,48	0.48
	PŁACHOCKICH Daromimi Blanc	wine 15	0,43	0.41
	PŁACHOCKICH Hibia XIV	wine 16	0,49	0.49
	DE SAS Parus A	wine 17	0,15	0.13
	DE SAS Parus B	wine 18	0,55	0.52
	DE SAS Milvus	wine 19	0,27	0.24
	SREBRNA GÓRA Cuvee	wine 25	0,66	0.6
	ADORIA Riesling	wine 27	1,10	1.05
	ADORIA Chordonnay	wine 28	1,46	1.43
	TURNAU Solaris	wine 31	0,29	0.36
	MOZÓW Mozów1	wine 32	0,86	0,86
red	JAWOREK pinore XII	wine 1	0,35	0.24
	PATRIA Ckoral	wine 5	0,18	0.11
	EQUUS Magnesia Presttige	wine 6	0,51	0.22
	CHODOROWA Rondo	wine 11	0,18	0.14
	PŁACHOCKICH Geltrus XIV	wine 12	0,34	0,32
	DE SAS Regulus A	wine 20	0,21	0,24
	DE SAS Regulus B	wine 21	0,24	0.13
	SREBRNA GÓRA Regent	wine 22	0,16	0.17
	SREBRNA GÓRA Cabernet Cortis	wine 23	0,09	0.14
	SREBRNA GÓRA Rondo	wine 24	0,32	0.22
	SREBRNA GÓRA Pinot Noir	wine 26	0,42	0.48
	ADORIA Pinot Noir	wine 29	0,57	0.52
	TURNAU Cabernet	wine 30	0,22	0.18
	MOZÓW Mozów3	wine 33	0,27	0.21
